# Effects of the invasive plant *Xanthium strumarium* on diversity of native plant species: A competitive analysis approach in North and Northeast China

**DOI:** 10.1371/journal.pone.0228476

**Published:** 2020-11-19

**Authors:** Mazher Farid Iqbal, Ming-Chao Liu, Aafia Iram, Yu-Long Feng

**Affiliations:** 1 Liaoning Key Laboratory for Biological Invasions and Global Changes, College of Bioscience and Biotechnology, Shenyang Agricultural University, Shenyang, Liaoning Province, China; 2 Government of the Punjab Education Department, Gujranwala, Punjab, Pakistan; Shandong Normal University, CHINA

## Abstract

*Xanthium strumarium* is native to North America and now has become one of the invasive alien species (IAS) in China. In order to detect the effects of the invader on biodiversity and evaluate its suitable habitats and ecological distribution, we investigated the abundance, relative abundance, diversity indices, and the number of the invasive and native plants in paired invaded and non-invaded quadrats in four locations in North and Northeast China. We also analyzed the effects of monthly mean maximum and minimum temperatures, relative humidity (%), and precipitations (mm). Strong positive significant (*P* < 0.01) correlation and maximum interspecific competition (41%) were found in Huailai between invaded and non-invaded quadrats. Shannon’s Diversity Index showed that non-invaded plots had significantly (*P* < 0.05) more diversified species than invaded ones. The significant (*P* < 0.05) Margalef’s Richness Index was found in Huailai and Zhangjiakou in non-invaded recorded heterogeneous nature of plant communities. Similarly, significant (*P* < 0.05) species richness found in Huailai and Zhangjiakou in non-invaded quadrats compared to invaded ones. Maximum evenness of *Setaria feberi* (0.47, 0.37), *Seteria viridis* (0.43) found in Fushun and Zhangjiakou recorded more stable in a community compared to other localities. Evenness showed positive relationship of Shannon Entropy within different plant species. The higher dissimilarity in plant communities found in Huailai (87.06%) followed by Yangyuan (44.43%), Zhangjiakou (40.13%) and Fushun (29.02%). The significant (*P* < 0.01) value of global statistics *R* (0.943/94.3%) showed high species diversity recorded in Huailai followed by Zhangjiakou recorded by non-metric multidimensional scaling and analysis of similarity between invaded and non-invaded plots. At the end it was concluded that the diversity indices reduced significantly (*P* < 0.05) in invaded quadrats indicated that native plant species become less diverse due to *X*. *strumarium* invasion. The degrees of *X*. *strumarium* invasion affected on species richness resulted to reduce diversity indices significantly in invaded quadrats.

## Introduction

Invasive plants are a major threat to the biodiversity and functioning of ecological systems [1–3]. Thus, biological invasions have become one of the hotspots in ecological research [4], The invasive plant *Xanthium strumarium* L. competes with native plant species for soil nutrients, moisture, “shelter”, “light”, and other resources, severely influencing natural vegetations [5]. The invasive plant is monoecious and annual herb of Asteraceae family, with broad leaves and tap-roots [6, 7]. It is native to North América and Argentina [8–10], but now become a noxious invader in China due to its strong colonization potential in new areas [11]. It has strong ability to adapt in diverse soils and climates. This plant has spread in six provinces in China including Hebei and Liaoning.

Invasive alien species has been recognized cause of biodiversity loss in the world [12]. *X*. *strumarium* has become a major concern in China, posing severe problems on the rangeland biodiversity, agriculture lands, parks, banks of river, and lakes, dams, roadsides, and even in urban areas, with great economic and ecological consequences. This plant is a severe threat to agriculture field crops such as soybeans, cotton, maize, sunflower and groundnuts in many parts of the world along with China [13]. It can also invade pastures and grazing lands causing reductions in forage production and fur damage through the thorns on the fruits [14]. It competes with, or even out-competes native species, decreasing genetic and species diversity within populations and altering ecosystem [15]. Multivariate analysis for ordination and analysis of similarity showed decreases in biodiversity indices in invaded over control sites, indicating that plant communities become less diverse due to *X*. *strumarium* invasion [16]. This plant has imposed negative impacts on ecosystem properties [17, 18].

**Fig 1:**
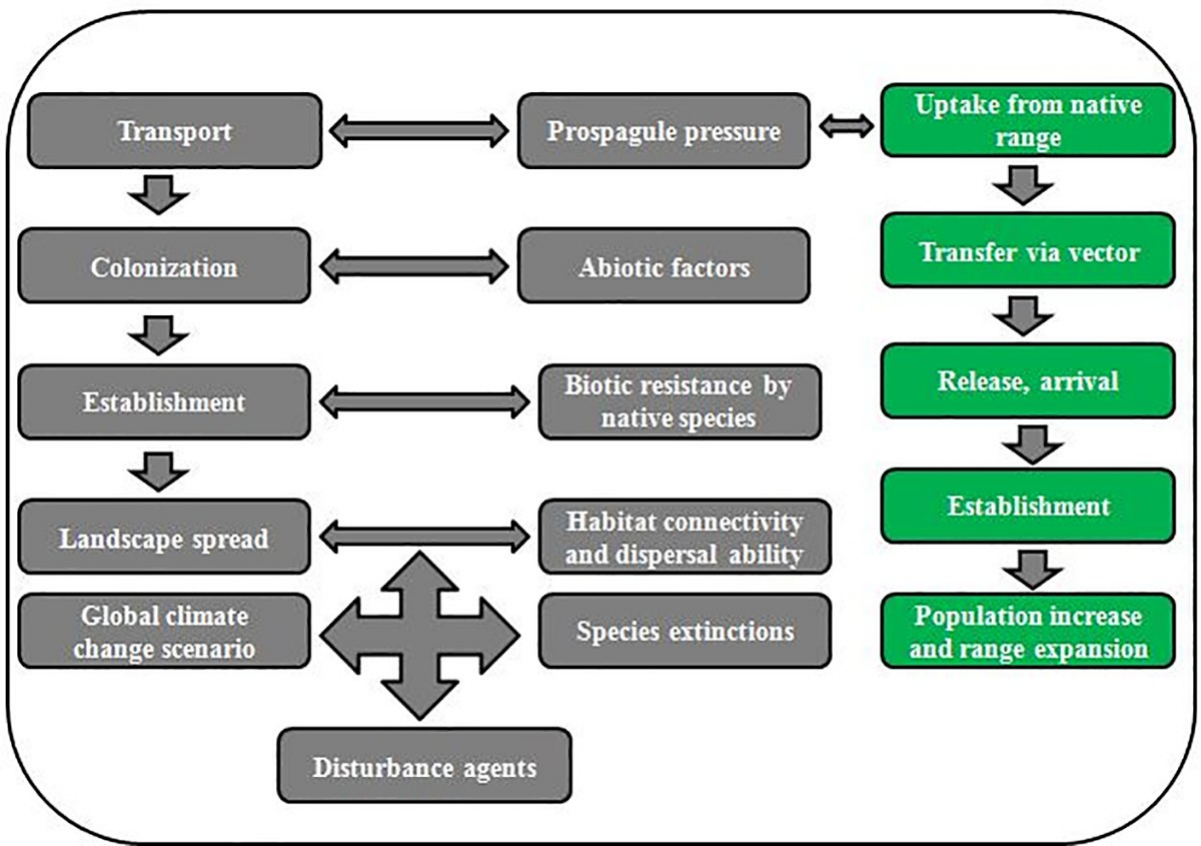
Main stages and factors affecting invasion success of introduced plants [20, 21].

The competitive patterns between invasive and native plant species may be different at different invasion steps such as transport stage, colonization stage, establishment stage, landscape spread stage (Fig 1) involved in invasion success [19].

*X*. *strumarium* usually competes with native plant species, and cause great loss in biodiversity. In order to detect the effects of the invader on biodiversity and evaluate its suitable habitats and ecological distribution, we investigated the abundance, relative abundance, diversity indices, and the number of the invasive and native plants in ten pairs of invaded and non-invaded quadrats in four locations in North and Northeast China (S1 Table in S1 File). In this study, we want to explore the following questions. (1) Does *X*. *strumarium* have significant effects on native plant species in paired comparison with diversity indices? (2) What is the paired correlation of different plant species both invaded and non-invaded quadrats in beta diversity? (3) How does *X*. *strumarium* invade and compete with native plant species in a paired comparison? (4) What is the relationship of Shannon Entropy with species diversity and evenness?

## Materials and methods

This study was carried out in Hebei and Liaoning Provinces in the summer (August and September) of 2018 using an ecological line transects method [22]. Three locations were selected in Hebei, i.e Yangyuan (40° 12′ 749″ N, 114° 39′ 920″ *E*), Huailai (40° 22′ 411′′ N, 115° 31′ 581′′ *E*), and Zhangjiakou (40° 51′ 290′′ N, 114° 51′ 378′′ *E*) and one location was selected in Liaoning, i.e., Fushun (41° 51′ 279′′ N, 123° 49′ 126′′ *E*). The precipitation is higher but monthly mean temperatures are lower in Fushun than in Zhangjiakou, Huailai and Yangyuan (S1 Fig in S1 File). In each location, 10 places were selected (10 replicates per location); and at each place, three pairs of invaded and non-invaded quadrats (100 * 100 cm) were setup (3 replicates per place). The 10 spaces in each location were at least 20 km apart from each other [23]. In order to diminish potential confounding effects of habitat heterogeneity on comparisons between invaded and non-invaded quadrats (i.e., the effects of the invader), the quadrats with and without *X*. *strumarium* were less than 2 m apart in each pair, and the quadrats in different pair were spaced at least 5 m [23]. The non-invaded quadrats were setup at second vegetations, and the invaded quadrats were dominated by *X*. *strumarium* [24]. In total, we had 120 pairs of quadrats with and without *X*. *strumarium* (4 locations × 10 places × 3 replicates). All plant species were identified and their individuals were counted in all invaded and non-invaded studied quadrats (S1 Table in S1 File) along with plant species did not found in studied quadrats but also found in four locations (S2 Table in S1 File). The averages of monthly mean maximum and minimum temperatures (Max. T and Mini. T, respectively), monthly mean precipitation (*PPT/ppt*) and monthly mean relative humidity (*RH*) was collected from data base of http://data.cma.cn/data/weatherBK.html (S1 Fig in S1 File).

Non-metric multidimensional scaling and analysis of similarity were conducted to determine the resemblance of species composition between the invaded and non-invaded quadrats through Bray-Curtis similarity/dissimilarity following log-transformation of plant species abundance data due to zero/no species count in some plots using one way analysis of variance with Global statistics *R* through PRIMER 7 programming [25]. The estimations of global measurement (*R*) values vary between +1 and -1; and the bigger of the *R* value is (close to 1), the more significant the dissimilarity (*P* < 0.01 or 1%) between the invaded and non-invaded quadrats is 0 to 100 with 100 expressing the most extreme dissimilarity [10, 26]. Similarity percentage method was used to assess contribution of each species to the dissimilarity between the invaded and non-invaded quadrats [27]. The degree of invasion impact was evaluated using diversity indices including Margalef's Richness Index, Shannon Entropy, Simpson’s Diversity Index, Shannon’s Diversity Index, Species Richness, Species Equitability or Evenness, and Abundance of different plant species between invaded and non-invaded quadrats [25, 28]. The differences in the diversity indices between the invaded and non-invaded quadrats were determined by analysis of variance with invasion status and locations as a factor in SPSS 13.0 (SPSS Inc., Chicago, IL, USA) and using Permutation analysis of variance through PRIMER 7 software. The competitive pattern was recorded between plant species within the selected quadrats calculated on the basis of Relative Abundance [25, 28–30]. The difference in plant species in each location between invaded, non-invaded quadrats and their correlation were calculated individually by paired *t*-test through SPSS 13.0 (SPSS Inc., Chicago, IL, USA).

## Results

Twelve plant species from ten genus and eight families were identified in our study, eight forbs, four grasses. Only two species (*Xanthium strumarium* and *Chenopodium album*) occurred in all four locations, with three species (*Artemisia annua*, *Setaria feberi*, and *Setaria viridis*) in three locations, two in two locations, and five in only one location (S1 Table in S1 File).

Some plant species were identified during field survey but not present in the selected quadrats. This data found that only one plant species found in three locations, eleven found in two locations and fifteen found in only one location, seventeen forbs, one sedge, three shrubs and six grass plant species (S2 Table in S1 File).

Shannon Entropy recorded in *Malva verticilata* (0.36) and *Ch*. *album* (0.36) with maximum evenness found in *Avena sativa* (0.20) and *S*. *viridis* (0.20) in its invaded quadrats were more stable in the community followed by other plant species. *M*. *verticilata* (0.94) and *Ch*. *album* (0.95) were more diversified in a community in its non-invaded quadrats at Yangyuan (S3A Table in S1 File). The calculated values of Shannon Entropy recorded high in *S*. *viridis* (0.33), *Cynunchum chinensis* (0.32); however, similar evenness was found by *Ch*. *album* (0.15) and *S*. *viridis* (0.15) in its invaded quadrats. *X*. *strumarium* was more diversified in the community at Huailai (0.74) compared to all other locations. Similarly, *S*. *feberi* (0.32), *Ch*. *album* (0.30), *A*. *annua* (0.30) found maximum Shannon Entropy with maximum evenness (0.15, 0.14, 0.14) was recorded that gave better stability in the community in its non-invaded quadrats at Huailai (S3B Table in S1 File). More Shannon Entropy was recorded by *S*. *feberi* (0.87) found with high evenness (0.37) recorded more diversified and stable in the community in its non-invaded quadrats at Zhangjiakou. Similar results were recorded with high diversity of *S*. *feberi* (0.47) and *S*. *viridis* (0.43) in its non-invaded quadrats at Fushun (S3C and S3D Table in S1 File).

**Fig 2:**
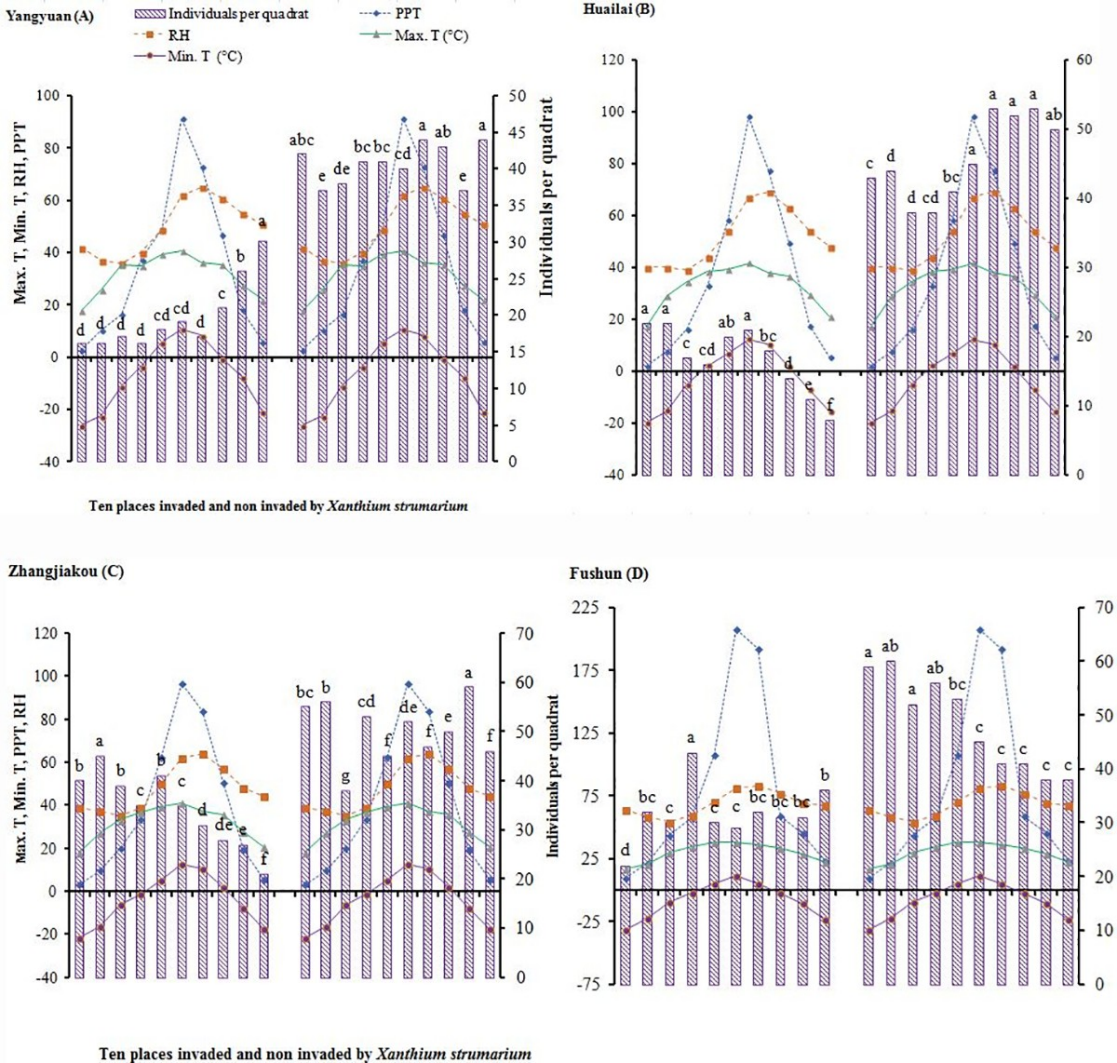
Number of plant individuals per quadrat (*N* = 10) and environmental factors (from February to November in 2018) found at ten invaded and ten non-invaded quadrats in Yangyuan (A), Huailai (B), Zhangjiakou (C) and Fushun (D), respectively. Max. T., average of monthly mean maximum temperature; Mini. T., average of monthly mean minimum temperature; RH, average of monthly mean relative humidity; PPT, average of monthly mean precipitation. Different letters indicates significant difference (*P* < 0.05; analysis of variance).

Strong significant (*P* < 0.01) correlation was recorded among invaded and non-invaded quadrats found in Huailai comparable to other locations showed nonsignificant (*P* > 0.05) investigations. Species richness recorded highly significant (*P* < 0.001) results in all locations. Native plant species are more diversified found significant (*P* < 0.05) individuals per quadrat in its non-invaded compared to invaded by *X*. *strumarium* (Table 1 and Fig 2).

**Table 1:** Analysis of variance of the invasion impact on species richness among invaded and non-invaded quadrats at different locations.

Locations	Invaded quadrat (Mean+SE)	Non-Invaded quadrat (Mean+SE)	Correlation of I versus NI	Species Richness
Yangyuan	19.60+0.86	40.71+0.49	N	***
Huailai	12.68+0.64	34.29+0.82	**	***
Zhangjiakou	34.20+1.32	50.09+1.10	N	***
Fushun	37.92+1.17	57.96+1.91	N	***

Whereas *N* = 30 (Number of quadrats 10 x 3 replication), *I* (Invaded), *NI* (Non-invaded); Mean + 1 *SE*

**, *P* < 0.01

***, *P* < 0.001, N, *P* > 0.05.

**Fig 3:**
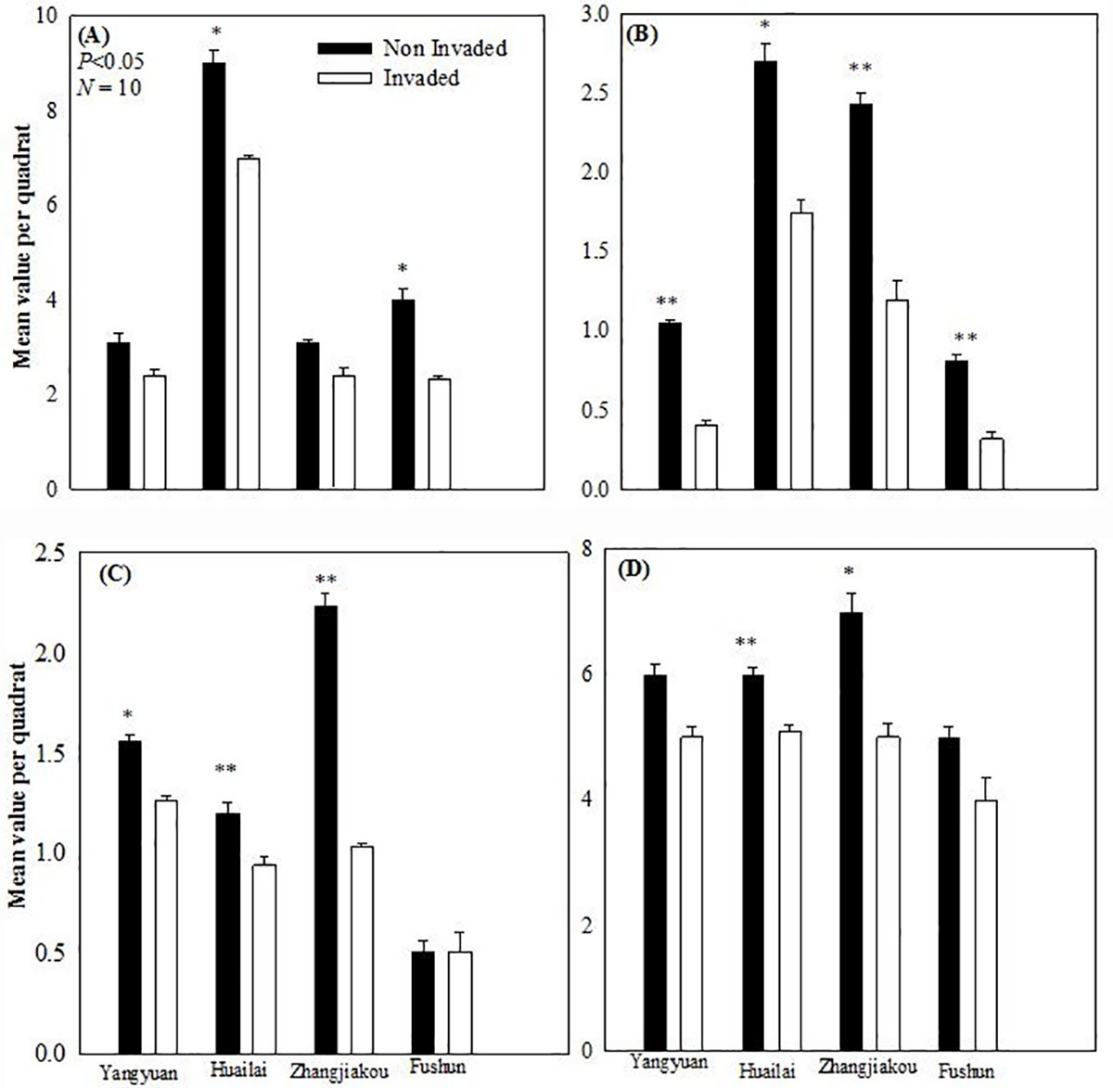
Difference in each ecological index between the invaded and non-invaded quadrats at Yangyuan, Huailai, Zhangjiakou and Fushun. (A), Abundance; (B), Shannon’s Diversity Index; (C), Margalef’s Richness Index; (D), Species Richness. Mean ± 1 *SE* (*N* = 10). *, *P* < 0.05, **, *P* < 0.01, NS, *P* > 0.05.

Significantly higher (*P* < 0.05) species richness was recorded at non-invaded quadrats in Zhangjiakou, Huailai and Yangyuan (Fig 3D). Significantly higher (*P* < 0.05) paired abundance (Fig 3A) was found in Huailai (*t* = 18.45, *P* = 0.003), Zhangjiakou (*t* = 7.56, *P* = 0.017) compared to Fushun (*t* = 2.18, *P* = 0.161). Strong positive nonsignificant (*P* = 0.281) paired samples correlations (*r* = 0.904) were recorded in Huailai. Shannon’s Diversity Index was significantly higher (*P* < 0.05) in non-invaded quadrats at Yangyuan (*t* = 13.86, *P* = 0.005), Huailai (*t* = 4.84, *P* = 0.040), Zhangjiakou (*t* = 30.44, *P* = 0.001), and Fushun (*t* = 10.61, *P* = 0.009) described in Fig 3B. Similarly highly significant (*P* < 0.01) Margalef's Richness Index found at Zhangjiakou (*t* = 23.09, *P* = 0.002), Huailai (*t* = 22.52, *P* = 0.002), Yangyuan (*t* = 5.20, *P* = 0.035), however nonsignificant (*P* > 0.05) index found in Fushun in both quadrats (*t* = 0.00, *P* = 1). There is a strong positive paired correlation (*r* = 1) and highly significant (*P* = 0.000) Margalef’s Richness Index found in Huailai and Zhangjiakou but strong negative and significant paired sampled correlation recorded in Yangyuan compared to moderate positive correlation (*r* = 0.5) recorded nonsignificant (*P* > 0.05) relationship at Fushun (Fig 3C).

**Fig 4:**
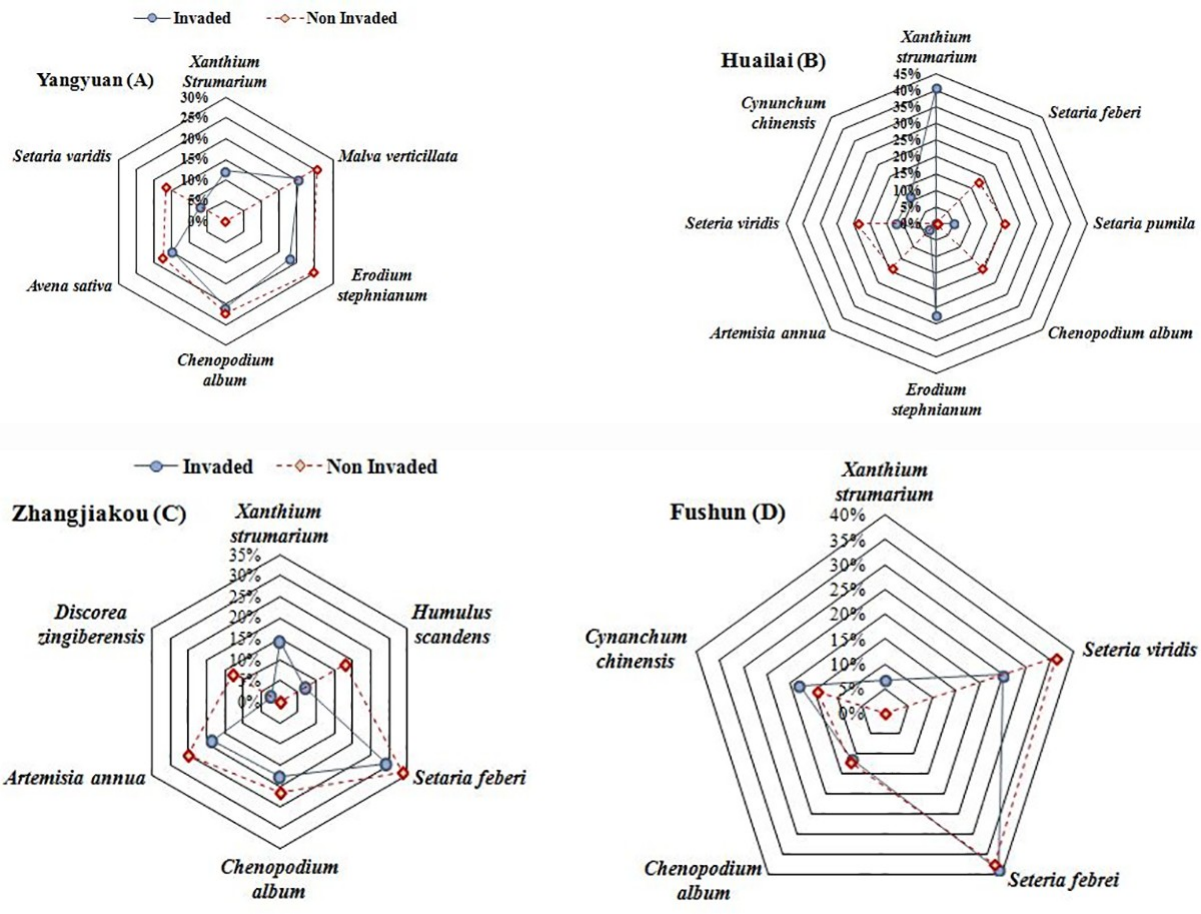
Web plot showed the interspecific competition between different plant species in invaded and non-invaded quadrats based on relative abundance. The central point is 0%, each ring gives the indication of the magnitude (%) of interspecific competition among plants in invaded and non-invaded quadrats.

In all four locations, interspecific competition intensity was significantly stronger (*P* < 0.05) at invaded relative to non-invaded quadrats. Species richness was significantly higher in non-invaded compared to invaded quadrat in Zhangjiakou (83.49 vs. 57.00), Huailai (57.15 vs. 21.14), Yangyuan (67.83 vs. 32.67), and Fushun (96.60 vs. 63.20) described in Fig 4. Maximum interspecific competition was found in invaded quadrats at Huailai (41%; Fig 4B), which indicates that *X*. *strumarium* competes with native plant species greatly in studied ecosystems. However, 14% and 12% interspecific competitions were found at invaded quadrats in Zhangjiakou and Yangyuan, respectively (Fig 4A and 4C). In Zhangjiakou *S*. *feberi* (34%), in Fushun *S*. *feberi* and *S*. *viridis* recorded maximum relative abundance in its non-invaded quadrats (Fig 4C and 4D).

Similarity percentage analysis suggested that abundance of plant species was high in non-invaded relative to invaded quadrats at four locations and their dissimilarities between the non-invaded and invaded quadrats were recorded significant investigations (Table 2).

**Table 2:** Abundances of the plant species present in each location, their dissimilarities (based on abundance) between the invaded and non-invaded quadrats, and their contributions (%) to the total dissimilarities.

Locations/Plant species	Average abundance in invaded quadrats*	Average abundance in non-invaded quadrats*	Average dissimilarity between invaded and non-invaded quadrats		Standard deviation	Contribution to dissimilarity (%)
**Yangyuan**						
*Malva verticilata*	4.00	10.40	10.64		2.54	23.94
*Seteria viridis*	1.40	6.80	9.24		1.95	20.80
*Chenopodium album*	4.10	9.00	8.27		2.27	18.62
*Avena sativa*	2.90	7.10	7.38		1.48	16.62
**Huailai**						
*Seteria viridis*	2.10	7.60		12.19	1.29	14.00
*Chenopodium album*	0.00	6.10		12.05	2.14	13.84
*Artemisia annua*	0.50	6.10		11.53	1.69	13.24
*Setaria pumila*	0.90	6.50		11.46	1.78	13.16
**Zhangjiakou**						
*Seteria febrei*	10.00	16.80		9.79	1.30	24.40
*Discorea zingiberensis*	0.80	6.50		7.28	1.44	18.13
*Artemisia annua*	6.30	12.50		7.27	1.81	18.12
**Fushun**						
*Seteria viridis*	7.90	17.50		11.84	2.41	40.82
*Seteria febrei*	12.30	8.10		7.86	1.47	27.10
*Chenopodium album*	3.60	5.80		4.31	1.58	14.86

The plant species collectively explained 79.98%, 54.24%, 60.65% and 82.78% of the total dissimilarities in Yangyuan, Huailai, Zhangjiakou and Fushun, respectively. The total dissimilarities were 44.43, 87.06, 40.13 and 29.02 in Yangyuan, Huailai, Zhangjiakou and Fushun, respectively.

Whereas

*: *1*-rare, *2*-common, *3*-very common, >*4*-dominant.

The total dissimilarities between non-invaded and invaded quadrats were 44.43, 87.06, 40.13, and 29.02 in Yangyuan, Huailai, Zhangjiakou, and Fushun, respectively. The most commonly occurring species collectively explained 79.98%, 54.24%, 60.65%, and 82.78% of the total dissimilarities found in Yangyuan, Huailai, Zhangjiakou, and Fushun, respectively. According to non-metric multidimensional scaling ordination and similarity analysis, the differences in species compositions between invaded and non-invaded quadrats were also significant, with global statistics *R* values found 0.62 (*P* < 0.01), 0.943 (*P* < 0.01), 0.779 (*P* < 0.01), and 0.553 (*P* < 0.01) in Yangyuan, Huailai, Zhangjiakou, and Fushun, respectively (S2 Fig in S1 File).

## Discussion

We examined mechanism of *Xanthium strumarium* invasion on native plant species found interspecific competition. *X*. *strumarium* affected directly with native plant species in its invaded quadrats. In Huailai, there was significant (*P* < 0.05) result among native species found in non-invaded quadrats, however maximum interspecific competition found in invaded quadrats at Huailai (41%). The decreased value of Shannon’s Diversity Index in invaded plot over non-invaded provided a clear indication that native plant species were less diversified due to *X*. *strumarium* invasion. In the non-invaded plots, the competition mechanism among native plants was significantly higher than invaded plots. The high Margalef's Richness Index in non-invaded found heterogeneous nature of plant communities. The native plant abundance reduced significantly (*P* < 0.05) in invaded plots due to *X*. *strumarium* invasion. These results are consistent with scientists who reported negative impact of *X*. *strumarium* on native plant species [16]. In our study 24 vs. 19 plant individuals present in all non-invaded vs. invaded quadrats are in line with the researchers who reported 70 vs. 31 plant species [25]. Margalef’s Richness Index and Shannon’s Diversity Index significantly (*P* < 0.05) decreased in the invaded plots up to 70.33% and 69.39% due to *X*. *strumarium* invasion [25]. A significant (*P*<0.05) Shannon’s diversity index recorded between non-invaded and invaded plots at Yangyuan, Huailai and Zhangjiakou. *X*. *strumarium* invasion mechanism found diversified in the community at Huailai recorded Simpson’s Diversity index (0.74) compared to all other locations. More Shannon Entropy was recorded by *Setaria feberi* (0.87) found with high evenness (0.37) recorded more diversified and stable in the community in its non-invaded quadrats at Zhangjiakou. Similar results were recorded with high diversity of *Seteria feberi* (0.47) and *Seteria viridis* (0.43) in its non-invaded quadrats at Fushun. Total number of individuals recorded in ten Invaded quadrats (169) compared to non-invaded (457) found strong positive significant correlation (*P* < 0.01) indicated *X*. *strumarium* invasion mechanism in Huailai. There is a positive relationship of Shannon Entropy with species diversity and evenness, however, species evenness increased with the increase of species diversity and Shannon Entropy. Shannon Entropy showed that different plant species in a community had a relative abundance and diverse in a community. These results are in line with the researchers who gave similar recommendations in their experiments [31–33]. The degrees of *X*. *strumarium* invasion affected on species richness in invaded quadrat resulted to reduce Shannon’s diversity index significantly (*P* < 0.05) are in line with the researchers who gave similar recommendations [34]. The native plant species abundance decreased significantly (P<0.05) up to 55.71% due to *X*. *strumarium* invasion. The researchers reported that twenty four families were recorded in non-invaded plots compared to fifteen in *X*. *strumarium* invaded areas. However, *X*. *strumarium* categorized as one of the dominant invasive alien plant that reduced native species and composition of various plant species in invaded communities [35]. The findings of our analysis are also consistent with the researchers who investigated strong impact of invaded species on native populations [36]. The diversity indices reduced in invaded plots compared to non-invaded supported our hypotheses that *X*. *strumarium* competed with native plant species because of their high phenotypic plasticity in the environment. These results are consistent with researchers who reported that *X*. *strumarium* showed its toxic effect on native plant populations in ecosystem [17]. A large canopy and tap root system of *X*. *strumarium* showed substantial impact on native plant communities, but its population abundance threatens native plant communities seriously. Invaded quadrats were found maximum uniformity and good competitor in ecosystem process. The plant invasions recorded serious influence on vegetation size and composition by interfering with biotic interactions in abiotic networks [37]. Maximum families (Asteraceae, Poaceae and Apocynaceae) found in our study during survey at different locations; however these three families have maximum tendency to flourish in an ecosystem (S2 Table in S1 File). Invasive species are characterized by their large, persistent, elastic, rapid growth, their ability to travel through vast areas and their rich reproduction allowed them to compete with indigenous plant species [38, 39]. The scientists recorded that the *X*. *strumarium* was the most aggressive alien-invasive plant in Ethiopia [40]. In invaded and non-invaded squares, researchers previously conducted paired comparative study which indicated that invaded plant species were competed and affected on native plant species [41]. The hypotheses are true that invasive alien plant can compete in a population with native plant species. These findings were consistent with researchers in their experiments who identified heterogeneous plant populations [42]. Maximum dissimilarity or beta diversity was recorded in Huailai (87.06%) followed by Yangyuan (44.43%), Zhangjiakou (40.13%), and Fushun (29.02%). *X*. *strumarium* influenced on species abundance in this study which was consistent with the researchers who identified Jaccard's similarity indices between invaded and non-invaded ranges that suggested loss of approximately 38.40% of plant species due to the *X*. *strumarium* invasions resulted in a 61.60% dissimilarity index [35]. Significantly (*P*<0.01) maximum global test for sample statistics *R* values were reported in Huailai (0.943/94.3%) followed by Zhangjiakou, Yangyuan and Fushun, where similarities were analyzed by one-way analysis of variance taking 999 number of permutations. The higher value of global statistics *R* suggested that there was a greater impact of species diversity on the variables studied. Yangyuan, Huailai, and Zhangjiakou areas were affected significantly by *X*. *strumarium* invasions. The lowest invasion was found in Fushun due to the high dissimilarity or beta diversity in invaded and non-invaded plots. Strong negative and significant (*P*<0.05) relationship (*R* = 0.741/74.1%, *P*<0.05) found between *X*. *strumarium* and species richness. These results are consistent with the scientists who stated that the regression equation and the Pearson correlation (-0.861) suggested the presence of negative linear relationship between *X*. *strumarium* and native species however, richness decreased significantly with the increased impact of *X*. *strumarium* [35].

## Conclusion

*Xanthium strumarium* was a good competitor against natural flora in the studied network. The elevated Shannon’s Diversity Index showed that plant networks in non-invaded areas were increased heterogeneously. *X*. *strumarium* invasion mechanism was diversified in the community at Huailai. Shannon Entropy recorded by *Setaria feberi* found high evenness were found more diversified and stable in the community in its non-invaded quadrats at Zhangjiakou. Similar findings were recorded and found high diversity of *Seteria feberi* and *Seteria viridis* at Fushun. Higher estimates of global statistics *R* showed the increased influence of decent species on the factors studied. The most affected location identified by *X*. *strumarium* invasion was Huailai followed by Zhangjiakou and Yangyuan. The mechanism of *X*. *strumarium* in our study recorded interspecific competition which suggested that this plant influenced negatively on native plant species. The degrees of *X*. *strumarium* invasion can affect species richness in invaded quadrat, resulting to reduce diversity indices significantly. There is dire need to develop integrated invasive plant management strategies to overcome *X*. *strumarium* invasion. This invasive plant species are spreading in cropped area of China, and may become future risk for growers. The study also encouraged researchers to explore the mechanism of protection, nutrient cycling, the role of pseudomonas bacteria, mechanism of resistance of invasive and native plants in future.

## Supporting information

S1 File (DOCX)
