# Supplementary material for: Effects of the invasive plant Xanthium strumarium on diversity of native plant species: A competitive analysis approach in North and Northeast China
Source: PLoS One. 2020 Nov 19;15(11):e0228476. doi: 10.1371/journal.pone.0228476 (PMC7676722; doi:10.1371/journal.pone.0228476)
Supplement: S1 File — (DOCX) [file pone.0228476.s001.docx]

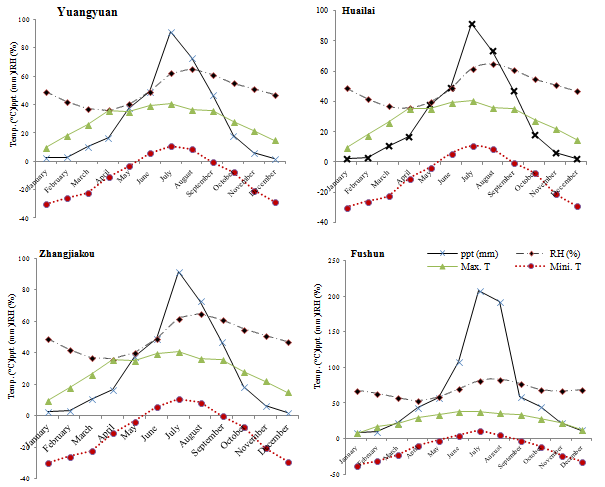


**S1 Fig.** Monthly climatic data of different locations in Liaoning and Hebei Provinces for the year 2018. ppt, precipitation/rainfall in mm; RH, relative humidity in %; Max. T, monthly mean maximum temperature; Mini. T, monthly mean minimum temperature.


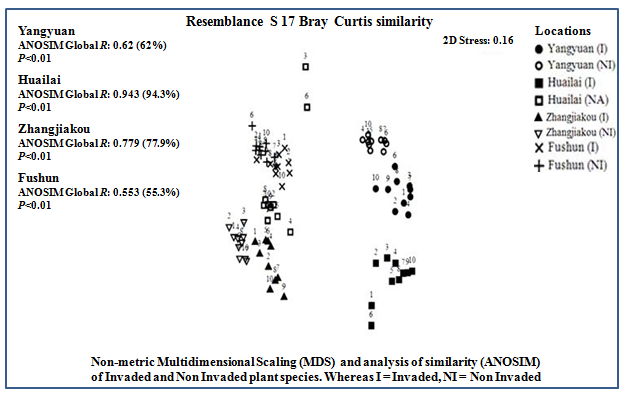


**S2 Fig.** Non-metric multidimensional scaling (nMDS) and analysis of similarity (ANOSIM) ordination plot showing differences between invaded and non-invaded quandrats at different locations in Liaoning and Hebei Provinces. Whereas aerobic number in the figure indicated the number of quadrats studied both invaded and non invaded plots.

**S1 Table.** Plant species studied during field survey with name of families, name of species and life forms at four locations.

| **Name of families** | **Name of species** | **Life forms** | **Yangyuan**  **(40°12.749 N / 114°39.920 E, 900 m)** | **Huailai**  **(40°22.411 N / 115°31.581 E, 1696 m)** | **Zhangjiakou (40°51.290 N / 114°51.378 E, 716 m)** | **Fushun (41°51.279 N / 123°49.126 E, 70 m)** |
| --- | --- | --- | --- | --- | --- | --- |
| Amaranthaceae | *Chenopodium album* | Forb | √ | √ | √ | √ |
| Apocynaceae | *Cynanchum chinensis* | Forb |  | √ |  |  |
| Asteraceae | *Artemisia annua* | Forb |  | √ | √ | √ |
| Asteraceae | *Xanthium strumarium* | Forb | √ | √ | √ | √ |
| Cannabaceae | *Humulus scandens* | Forb |  |  | √ | √ |
| Dioscoreaceae | *Dioscorea zingiberensis* | Forb |  |  | √ |  |
| Geraniaceae | *Erodium stephnianum* | Forb | √ | √ |  |  |
| Malvaceae | *Malva verticillata* | Forb | √ |  |  |  |
| Poaceae | *Avena sativa* | Grass | √ |  |  |  |
| Poaceae | *Setaria feberi* | Grass |  | √ | √ | √ |
| Poaceae | *Setaria pumila* | Grass |  | √ |  |  |
| Poaceae | *Seteria viridis* | Grass | √ | √ |  | √ |

Whereas *N* (North Latitude), *E* (East Longitude), *m* (Altitude in meter), ° (Degree).

**S2 Table.** Plant species identified but unstudied during field survey with name of families, species and life forms at four locations

| **Families** | **Botanical names** | **Life forms** | **Yangyuan**  **(40°12.749 N / 114°39.920 E, 900 m)** | **Huailai**  **(40°22.411 N / 115°31.581 E, 1696 m)** | **Zhangjiakou (40°51.290 N / 114°51.378 E, 716 m)** | **Fushun (41°51.279 N / 123°49.126 E, 70 m)** |
| --- | --- | --- | --- | --- | --- | --- |
| Amaranthaceae | *Amaranthus retroflexus* | Forbs |  | √ |  | √ |
| Amaranthaceae | *Amaranthus blitum* | Forbs | √ |  |  | √ |
| Amaranthaceae | *Chenopodium hybridum* | Forbs |  | √ |  | √ |
| Amaranthaceae | *Chenopodium ficifolium* | Forbs |  |  | √ | √ |
| Apocynaceae | *Cynanchum wilfordii* | Forbs |  | √ |  |  |
| Asteraceae | *Ambrosia trifida* | Forbs |  |  |  | √ |
| Asteraceae | *Ambrosia artemisifolia* | Forbs |  | √ |  | √ |
| Asteraceae | *Artemisia selengensis* | Forbs |  |  |  | √ |
| Asteraceae | *Erigeron acris* | Forbs | √ | √ |  | √ |
| Asteraceae | *Taraxacum mongolicum* | Forbs |  | √ |  |  |
| Caryophyllaceae | *Myosoton aquaticum* | Forbs |  | √ |  |  |
| Chenopodiaceae | *Kochia scoparia* | Forbs |  |  | √ |  |
| Cyperaceae | *Cyperus cyperoides* | Sedge |  |  |  | √ |
| Euphorbiaceae | *Acalypha australis* | Shrub |  |  |  | √ |
| Euphorbiaceae | *Euphorbia maculata* | Shrub |  |  |  | √ |
| Fabaceae | *Medicago lupulina* | Forbs | √ |  |  |  |
| Oxalidaceae | *Oxalis corniculata* | Shrub |  |  |  | √ |
| Plantaginaceae | *Plantago depressa* | Forbs | √ |  |  |  |
| Polygonaceae | *Polygonum aviculare* | Forbs |  |  |  | √ |
| Polygonaceae | *Polygonum longisetum* | Forbs |  |  |  | √ |
| Poaceae | *Axonopus compressus* | Grass | √ |  |  |  |
| Poaceae | *Echinochola crus-galli* | Grass | √ | √ |  |  |
| Poaceae | *Eleusine indica* | Grass |  | √ |  | √ |
| Poaceae | *Imperata cylindrica* | Grass | √ | √ |  |  |
| Poaceae | *Leptochloa chinensis* | Grass | √ | √ |  |  |
| Poaceae | *Zizania latifolia* | Grass | √ | √ |  |  |
| Rubiaceae | *Rubia cordifolia* | Forbs | √ | √ |  |  |

Whereas *N* (North Latitude), *E* (East Longitude), *m* (Altitude in meter), ° (Degree). Fifteen plant species present in one location, eleven species are found in two locations and only one plant species present in one location.

**S3 Table. Rank of diversity indices of different plant species collected from Invaded and Non Invaded quadrats from different localities (A) Yangyuan, (B) Huailai, (C) Zhangjiakou, (D) Fushun**

| **R** | **Plant Species** | **Species Diversity** | | | | | |
| --- | --- | --- | --- | --- | --- | --- | --- |
|  |  | **Invaded** | | | **Non Invaded** | | |
|  |  | **SE** | **SDI** | **E** | **SE** | **SDI** | **E** |
| (A) Yangyuan |  |  |  |  |  |  |  |
| 1 | *Xanthium strumarium* | 0.34 | 0.99 | 0.15 | 0 | 1 | 0 |
| 2 | *Erodium stephnianum* | 0.35 | 0.95 | 0.16 | 0.22 | 0.97 | 0.10 |
| 3 | *Malva verticillata* | 0.36 | 0.96 | 0.15 | 0.04 | 0.94 | 0.02 |
| 4 | *Chenopodium album* | 0.36 | 0.96 | 0.15 | 0.09 | 0.95 | 0.04 |
| 5 | *Avena sativa* | 0.35 | 0.98 | 0.20 | 0.24 | 0.97 | 0.10 |
| 6 | *Setaria viridis* | 0.35 | 0.98 | 0.20 | 0.26 | 0.97 | 0.11 |
| Species Richness | *Ni* | 2.11 |  |  | 0.85 |  |  |
|  | *N* | 5.1 |  |  | 5 |  |  |
|  | *R* | 1.11 | *E* |  | 0.78 | *E* |  |
|  | *d* | 4.77 | 1.31 |  | 4.73 | 0.48 |  |
| (B) Huailai |  |  |  |  |  |  |  |
| 1 | *Xanthium strumarium* | 0.25 | 0.74 | 0.10 | 0 | 1 | 0 |
| 2 | *Setaria pumila* | 0.22 | 0.99 | 0.20 | 0.28 | 0.98 | 0.13 |
| 3 | *Seteria feberi* | 0.11 | 0.99 | 0.00 | 0.32 | 0.98 | 0.15 |
| 4 | *Chenopodium album* | 0.24 | 0.99 | 0.15 | 0.3 | 0.98 | 0.14 |
| 5 | *Artemisia annua* | 0.15 | 0.99 | 0.00 | 0.3 | 0.98 | 0.14 |
| 6 | *Seteria viridis* | 0.33 | 0.97 | 0.15 | 0.21 | 0.97 | 0.09 |
| 7 | *Cynunchum chinensis* | 0.32 | 0.98 | 0.18 | 0.19 | 0.97 | 0.08 |
| Species Richness | *Ni* | 1.62 |  |  | 1.60 |  |  |
|  | *N* | 3.4 |  |  | 5.3 |  |  |
|  | *R* | 1.01 | *E* |  | 0.94 | *E* |  |
|  | *d* | 2.98 | 1.31 |  | 5 | 0.95 |  |
| (C) Zhangjiakou |  |  |  |  |  |  |  |
| 1 | *Xanthium strumarium* | 0.35 | 0.98 | 0.15 | 0 | 1 | 0 |
| 2 | *Humulus scandens* | 0.3 | 0.97 | 0.15 | 0.37 | 0.99 | 0.18 |
| 3 | *Seteria febrei* | 0 | 0.91 | 0 | 0.87 | 0.89 | 0.37 |
| 4 | *Chenopodium album* | 0.3 | 0.97 | 0.13 | 0.07 | 0.95 | 0.03 |
| 5 | *Artemisia annua* | 0.29 | 0.97 | 0.13 | 0.28 | 0.94 | 0.12 |
| 6 | *Discorea zingiberensis* | 0.29 | 0.99 | 0.18 | 0.28 | 0.98 | 0.13 |
| Species Richness | *Ni* | 1.53 |  |  | 1.87 |  |  |
|  | *N* | 5.1 |  |  | 4.6 |  |  |
|  | *R* | 0.88 | *E* |  | 0.65 | *E* |  |
|  | *d* | 4.81 | 0.89 |  | 4.34 | 0.38 |  |
| (D) Fushun |  |  |  |  |  |  |  |
| 1 | *Xanthium strumarium* | 0.33 | 0.99 | 0.16 | 0 | 1 | 0 |
| 2 | *Chenopodium album* | 0.37 | 0.98 | 0.16 | 0.32 | 0.98 | 0.14 |
| 3 | *Seteria feberi* | 0.25 | 0.85 | 0.11 | 1.07 | 0.86 | 0.47 |
| 4 | *Cynunchum chinensis* | 0.32 | 0.97 | 0.14 | 0.26 | 0.98 | 0.11 |
| 5 | *Seteria viridis* | 0.19 | 0.94 | 0.08 | 0.98 | 0.87 | 0.43 |
| Species Richness | *Ni* | 1.46 |  |  | 2.63 |  |  |
|  | *N* | 4.8 |  |  | 3.9 |  |  |
|  | *R* | 0.86 | *E* |  | 0.57 | *E* |  |
|  | *d* | 4.51 | 0.6 |  | 3.64 | 1.09 |  |

*R* (Rank), *SE* (Shannon Entropy), *SDI* (Simpson’s Diversity Index), *E* (Evenness), *Ni* (Number of individuals), *N* (Number of species), *R* (Menhenick index), *d* (Margalef’s Index)
